# Case report: Solitary fibrous tumor of the paracervical uterus combined with vaginal wall adenocarcinoma

**DOI:** 10.3389/fmed.2024.1456221

**Published:** 2024-08-14

**Authors:** Xiaowei Zhang, Jun Chen, Junqiang Du, Jiajia Ying

**Affiliations:** ^1^Department of Pathology, Affiliated Dongyang Hospital of Wenzhou Medical University, Dongyang, Zhejiang, China; ^2^Department of Medical Imaging, Affiliated Dongyang Hospital of Wenzhou Medical University, Dongyang, Zhejiang, China; ^3^Department of Gynaecology, Affiliated Dongyang Hospital of Wenzhou Medical University, Dongyang, Zhejiang, China; ^4^Department of Surgical Center, Affiliated Dongyang Hospital of Wenzhou Medical University, Dongyang, Zhejiang, China

**Keywords:** solitary fibrous tumor, mesenchymal tumors, pleura, uterine cervix, vaginal wall adenocarcinoma, total hysterectomy

## Abstract

**Background:**

Solitary fibrous tumors are rare mesenchymal tumors that typically occur in the pleura. Solitary fibrous tumors of the uterine cervix are uncommon. We report the first case of a patient who underwent total hysterectomy for vaginal wall adenocarcinoma and was found to have a concurrent solitary fibrous tumor in the paracervical-uterus.

**Case presentation:**

A 51-year-old woman was admitted to our hospital due to contact bleeding. A gynecological examination revealed nodules of 3.0 × 1.0 cm on the vaginal wall, and a colposcopy with biopsy revealed adenocarcinoma of the vaginal wall. After the recommended staging examinations, the patient underwent total hysterectomy, double adnexectomy, pelvic lymph node dissection, and vaginal wall resection. During surgery, a nodule measuring approximately 2 × 2 cm was found in the middle of the mass in the left paracervical region. Subsequent postoperative histopathological examination confirmed an solitary fibrous tumor of the uterine cervix with adenocarcinoma of the vaginal wall. The patient was followed up for 46 months after hospitalization, and no recurrence or distant metastases were observed.

**Conclusion:**

In rare cases, solitary fibrous tumors may form large masses in the cervical or vaginal wall. They can easily be misdiagnosed as benign or malignant cervical tumors before and during surgery. Postoperative pathology and immunohistochemistry are helpful for diagnosis. Most solitary fibrous cervical tumors are benign, occasionally with low malignant potential, and surgical treatment is feasible and effective.

## 1 Introduction

Solitary fibrous tumors (SFTs) are rare mesenchymal tumors that usually occur in the visceral pleura. SFTs can develop anywhere in the body but rarely occur in the paracervical region. We believe that this is the first reported case of a solitary fibrous neoplasm in the uterine cervix combined with adenocarcinoma of the vaginal wall. This case report aimed to discuss the clinicopathological features of cervical SFTs to provide a reference for the clinical diagnosis and treatment of cervical tumors combined with vaginal wall neoplasms and to avoid missed diagnoses and misdiagnoses.

## 2 Case presentation

A 51-year-old woman was admitted to the hospital on March 13, 2020, due to postcoital contact bleeding. She had a history of regular menstruation with a 37-day cycle, a bleeding period of 7 days, moderate menstrual volumes, occasional dysmenorrhea, and natural menopause 6 months prior to presentation. Previous cervical cytology results indicated no abnormalities, and human papillomavirus tested negative. Following sexual intercourse, the patient experienced four episodes of bleeding, with no significant abdominal pain or distention reported during these episodes. Upon gynecological examination, nodules measuring 3.0 × 1.0 cm were observed on the cervical-vaginal wall. Colposcopy with biopsy was recommended, and histopathological examination indicated adenocarcinoma of the vaginal wall. Consequently, further surgery was advised. The patient had no history of intrauterine exposure to diethylstilbestrol.

### 2.1 Specialist physical examination

The patient was married and nulliparous. The results of the patient’s physical examination were as follows: the vulva was normal, and a nodule measuring approximately 3.0 × 1.0 cm was identified at 9 o’clock on the vaginal wall. The mass was hard and brown, displayed poor mobility, and was prone to bleeding upon touch. The surface of the cervix appeared smooth, with no discernible abnormalities. The uterus was anteriorly positioned, of average size and medium quality, mobile, and non-tender. Additionally, no conspicuous masses or tenderness were noted in the bilateral adnexal areas.

### 2.2 Ultrasound examination

Vaginal ultrasound revealed that the uterus was tilted forward and was normal in size. A low-echo nodule measuring 24 × 19 mm was detected on the posterior wall, characterized by unclear boundaries and an uneven internal echo.

### 2.3 Pelvic computed tomography

Pelvic computed tomography revealed a small amount of free gas following the biopsy of the vaginal tumor. A similar round isodensity shadow was observed on the left lateral wall of the cervix, which exhibited enhancement after contrast agent administration. Notably, no enlarged pelvic lymph nodes were observed.

### 2.4 Pelvic enhanced magnetic resonance imaging

**Figure 1:**
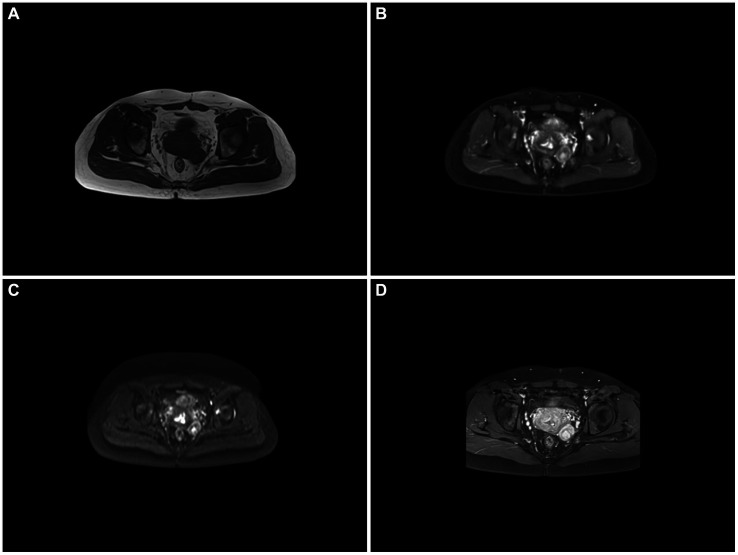
**(A–D)** Magnetic resonance imaging. The left posterior cervical tubercle is seen on a T1-weighted image (T1WI) with a slightly low signal, T2-weighted fat suppression (T2-FS) image with high and low mixed signals, and on diffusion-weighted imaging (DWI) with partially limited diffusion, showing enhancement after noticeable inhomogeneous enhancement, and visible snake vascular signs.

Pelvic-enhanced magnetic resonance imaging revealed the catheter filling the operative area following a biopsy of the right vaginal wall. No local vaginal wall thickening or surrounding fistulae was observed. However, there was an increase in uterine volume and uneven thickening of the uterine wall. A similar round mixed-signal shadow, approximately 3.4 × 2.6 cm in size, with a clear boundary, was observed near the left posterior wall of the cervix. Enhancement in this area displayed inhomogeneous enhancement. Notably, enlarged pelvic lymph nodes were not observed (Figure 1).

**Figure 2:**
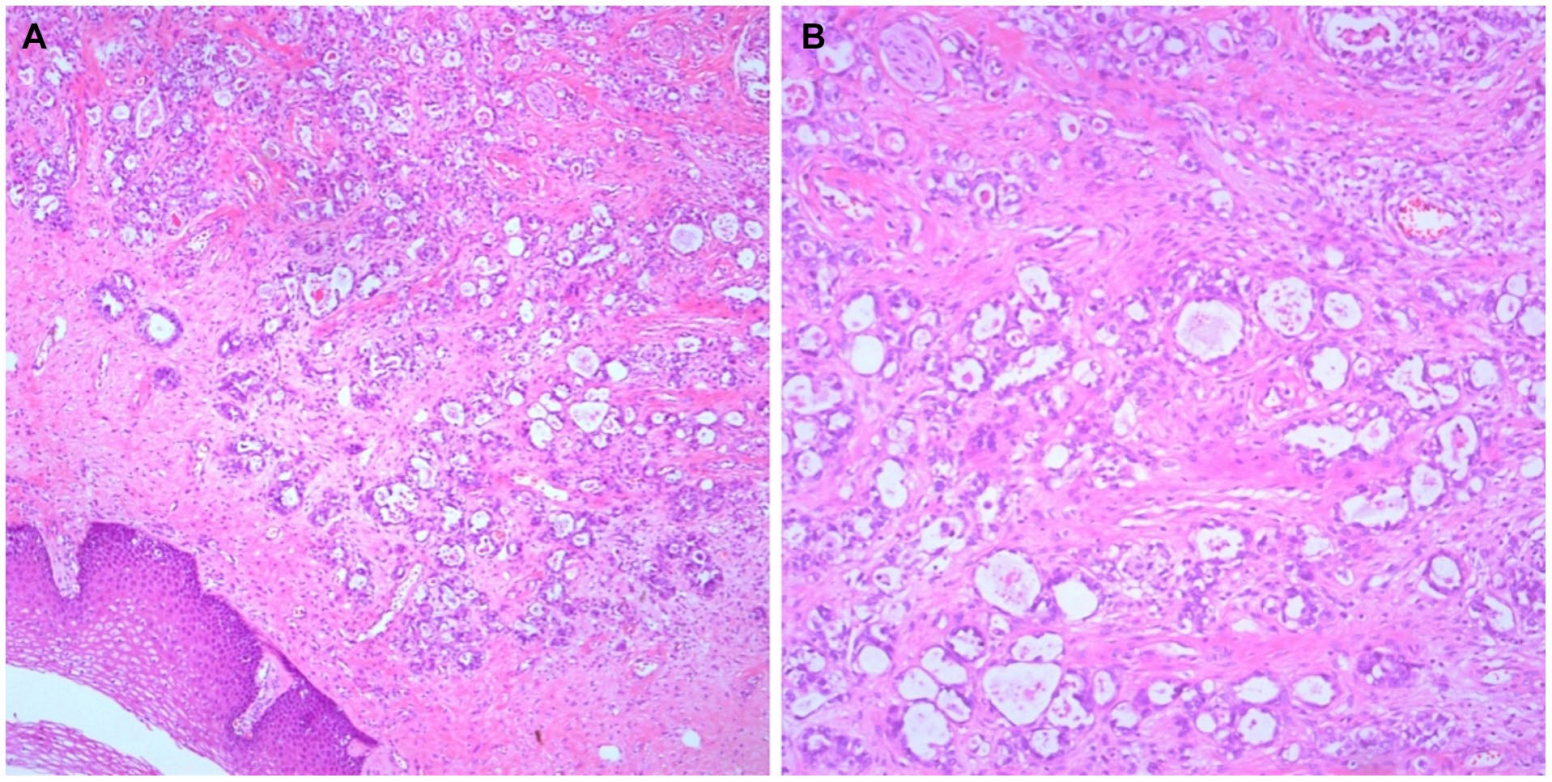
Histological findings. Adenocarcinoma can be seen on the vaginal wall with its typical morphological findings (**A**, ×50; **B**, ×100).

Based on the preoperative imaging findings, the preoperative International Federation of Gynecology and Obstetrics (FIGO) clinical stage was determined to be I. On March 15, 2020, a total hysterectomy, double adnexectomy, pelvic lymph node dissection, and vaginal wall resection were performed. Intraoperatively, the uterus was positioned anteriorly without bilateral paracentral thickening. Portions of the intestine and mesentery were found to be adherent to the posterior wall of the uterus and pelvic floor. No enlarged lymph nodes were identified in bilateral pelvic cavities or around the abdominal aorta. A mass measuring approximately 2 × 2 cm was discovered in the left-sided paracervical uteri. The postoperative histopathological report revealed clear cell adenocarcinoma of the vaginal wall involving the cervix and infiltrating the deep muscle layer. Additionally, a vascular thrombus was observed, whereas no obvious nerve involvement. Adenocarcinoma metastases were found in one of the 23 pelvic lymph nodes examined histopathologically. The vaginal wall incision margin was negative. These results and combined morphology and immunohistochemistry of the cervix uteri were consistent with those of an SFT. Excision of the vaginal wall yielded negative results (Figures 2A–C).

**Figure 3:**
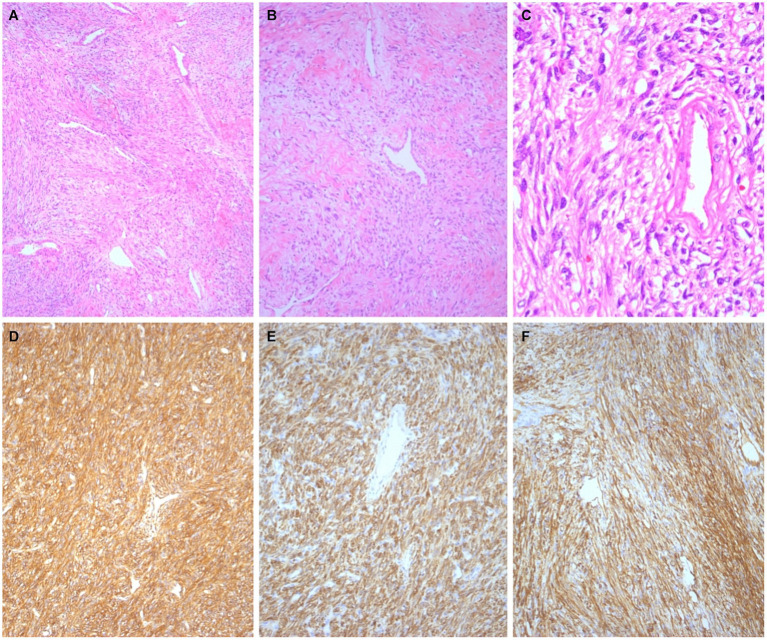
Histological findings. The tumor cells in the cervix exhibited oval to spindle-shaped morphology with patternless proliferative manifestations and prominent branching, thin-walled hemangiopericytoma vessels (**A**, ×50; **B**, ×100; **C**, ×400). Immunohistochemical staining revealed strong expression of CD99 (**D**, ×100), BCL-2 (**E**, ×100), and CD34 (**F**, ×100).

Immunohistochemical analysis of the cervical lesions revealed the following results: positive for vimentin and CD34 and negative for Cytokeratin, S-100, smooth muscle antigen, desmin, actin, estrogen receptors, and progesterone receptors. The Ki-67 proliferation index was 2%. Moreover, the SFT tumor cells were positive for CD99 and Bcl-2 (Figures 3D–F). Immunohistochemical analysis of the vaginal wall adenocarcinoma revealed the following results: positive for CK7 and CK8. The postoperative clinicopathological stage was determined to be T1N1M0, with a FIGO clinical stage of III. Postoperatively, the patient underwent chemotherapy with docetaxel and carboplatin administered 6 times, along with radiotherapy administered 25 times. The patient was followed up for 46 months after hospitalization, and no recurrence or distant metastases were observed.

## 3 Discussion

SFTs usually occur in the pleura. However, extrapleural SFTs can be found in various locations, including the head and neck, upper respiratory tract, mediastinum, pelvic cavity, retroperitoneum, and surrounding soft tissues. Meanwhile, these tumors can arise in almost all parts of the body, including the central nervous system, meninges, notochords, parotid glands, thyroid gland, lung parenchyma, liver, gastrointestinal tract, adrenal glands, bladder, prostate, spermatic cord, and testes. Although SFTs can occur in the female genital tract, they are infrequent in the cervix (1–4).

The histological origin of SFTs remains unclear. Previously, because SFTs were found mainly in the pleural cavity, it was thought that tumor cells differentiated from mesothelial cells into fibroblasts. However, recent immunohistochemical studies have revealed that tumor cells in SFTs lack mesothelial characteristics but express CD34 and Bcl-2, suggesting that the tumor originates from mesenchymal tissues (5–7). Therefore, SFTs are believed to originate from CD34+ dendritic mesenchymal cells, which have a morphology similar to that of fibroblasts. These cells are widely distributed in human connective tissues and can support and promote the proliferation and differentiation of adjacent stem cells (8).

Cervical SFTs are usually local, slow-growing, painless masses that are often detected accidentally. The clinical manifestations of cervical SFTs are easily misdiagnosed as cervical polyps (9). Owing to the lack of specific clinical manifestations in the early stages, most cases are discovered accidentally during imaging examinations. In some instances, a large mass in the cervix may be found, causing abnormal uterine bleeding. Although cervical SFTs can occur in patients between 5 and 87 years of age, they occur primarily in females between 30 and 60 years of age. Radiographically, they often appear as well-circumscribed round or oval masses.

Cervical SFTs are often misdiagnosed as more common tumors, including uterine leiomyomas or cervical cancer. A definitive diagnosis of an SFT of the cervix is challenging preoperatively, and the diagnosis relies on postoperative histopathological morphology and immunohistochemistry results. The pathological mass is usually round or oval with clear perimeters. In some cases, fibrous pseudo-envelopes that are tough and elastic and can exhibit mucoid degeneration line the mass (10). Histopathologically, our patient’s tumor showed clear circumscription of alternating areas with abundant and sparse cells. The tumor cells were short, spindle-shaped, or oval in the area, with abundant cells, little or unclear cytoplasm, and uniform nuclear chromatin.

Conversely, the tumor cells in the sparse areas were slender and spindle-shaped. The cells showed no obvious atypia in either region, and mitotic images were rare. The tumor cells contained collagen fibers of varying thicknesses and shapes. Abundant blood vessels are also present within these tumors, with collagen degeneration of the vascular wall observed in some instances. Furthermore, significant hyaline and mucoid degeneration may be present in the interstitium.

Immunohistochemistry of SFT cells in the female genital tract typically demonstrates the expression of CD34, Bcl-2, CD99, and STAT6. However, no expression of estrogen or progesterone receptors, actin, or desmin is usually seen (11).

### 3.1 Differential diagnosis

Cervical SFTs must be distinguished from other benign or malignant tumors that may occur in the cervix.

1. Cervical leiomyoma: Cervical smooth muscle tumors exhibit classic morphology with an interlacing arrangement pattern. Tumor cell cytoplasm stains red, with rod-shaped nuclei and smooth muscle features. Immunohistochemical staining is positive for smooth muscle antigen, h-Caldesmon, and desmin but negative for CD34, Bcl-2, CD99, and STAT6.
2. Cellular angiofibromas: Cellular angiofibromas are common in middle-aged and older adult females and are found in the vulva, groin, and vagina. The tumor border is clear and comprises oval-to-spindle proliferative cells often arranged in bundles. The cells are well-differentiated and lack nuclear mitotic figures. The tumor contains numerous small-to-medium-sized blood vessels, some of which may exhibit hyaline degeneration. The tumor cells do not express Bcl-2, CD99, or CD34, which is beneficial for differentiation.
3. Superficial cervicovaginal myofibroblastoma: Superficial cervicovaginal myofibroblastomas are mesenchymal tumors of the female vaginal or cervical mucosa, often with a polypoid appearance and jelly-like mucus. Microscopic findings show clear tumor boundaries, sparse components of tumor cells in the superficial area, mucinous or edematous interstitium, relatively dense tumor cells in the central area, more collagen fibers in the interstitium, and a small to moderate number of blood vessels. Tumor cells express desmin and CD34 but are negative for CD99, Bcl-2, and STAT6.
4. Angiomyofibroblastomas: Angiomyofibroblastomas predominantly occur in the vulva, with the perineum and vagina as secondary sites. These tumors may or may not have an capsule. Angiomyofibroblastomas are typically observed in young and middle-aged females. Microscopically, there are alternating areas of rich and loose cells comprising short fusiform or oval cells containing abundant thin-walled blood vessels. Tumor cells are often arranged in bundles around blood vessels, and areas with altered coloration may be observed. The immunophenotype is positive for vimentin and desmin but negative for CD34, Bcl-2 and CD99.
5. Sarcomatoid carcinoma: Sarcomatoid carcinoma of the cervix is very rare. These tumors comprise two components: an *in situ* or invasive squamous cell carcinoma and a malignant spindle cell component with mesenchymal morphology but expressing epithelial markers. This case was negative for cytokeratin and did not show cervical squamous cell carcinoma *in situ* or invasive squamous cell carcinoma.

Morphological changes in SFTs correlate with their biological behavior. Most histological manifestations of SFTs are inert, rarely recurrent, and usually do not metastasize but occasionally have low malignant potential. In cases of SFTs, the tumor should be entirely and locally resected, and the incisal margin should be negative (12). SFT recurrence is closely related to the completion of surgical resection. Rare recurrence is often caused by incomplete tumor resection. Therefore, special attention should be paid to the correct diagnosis and initial treatment (13). Our patient was diagnosed with vaginal wall adenocarcinoma, a rare malignant tumor that may occur sporadically or be associated with intrauterine exposure to diethylstilbestrol. Patients with vaginal adenocarcinoma usually present with abnormal vaginal bleeding, painful sexual intercourse, and clinically significant nodular or polypoid masses. In these patients, the adenocarcinoma can spread locally or metastasize lymphatically or hematologically, and its prognosis is related to the clinical stage. In the present case, the SFT was complicated by vaginal wall adenocarcinoma. Therefore, total hysterectomy, bilateral uterine adnexa resection, pelvic lymph node dissection, and vaginal wall resection were performed. Although SFTs are mesenchymal tumors and vaginal wall adenocarcinoma is an epithelial cancer, the two tumors are independent of each other. After the radical resection of the vaginal wall adenocarcinoma, only radiotherapy and chemotherapy were required for this case. Currently, multimodal approaches, including surgery, chemotherapy, and radiotherapy, appear to be safe and feasible for treating rare and aggressive neoplasms of the female genital tract (14).

Following the procedures, the postoperative clinicopathological stage was determined to be T1N1M0, with a FIGO clinical stage of III. The patient underwent follow-up examinations for 46 months after hospitalization, during which no recurrence or distant metastases were observed. For the postoperative follow-up of similar malignant tumors concomitant with benign or low malignant potential tumors, we recommend pelvic computed tomography or pelvic magnetic resonance imaging. Follow-up should be conducted at 3 months, 6 months, and 1 year postoperatively and then every 6 months thereafter.

## 4 Conclusion

Cervical SFTs are rare benign mesenchymal tumors,occasionally with low malignant potential that are easily misdiagnosed as other benign or malignant cervical tumors. We report the first case of a total hysterectomy for vaginal wall adenocarcinoma combined with uterine SFT, providing a clinical example of its diagnosis and treatment.

## Data availability statement

The original contributions presented in the study are included in the article/supplementary material, further inquiries can be directed to the corresponding author.

## Ethics statement

The studies involving humans were approved by Ethics Committee of Dongyang People’s Hospital. The studies were conducted in accordance with the local legislation and institutional requirements. Written informed consent for participation was not required from the participants or the participants’ legal guardians/next of kin in accordance with the national legislation and institutional requirements. Written informed consent was obtained from the individual(s) for the publication of any potentially identifiable images or data included in this article. Written informed consent was obtained from the participant/patient(s) for the publication of this case report.

## Author contributions

XZ: Conceptualization, Data curation, Formal analysis, Funding acquisition, Methodology, Project administration, Supervision, Visualization, Writing – original draft, Writing – review & editing. JC: Formal analysis, Investigation, Methodology, Project administration, Resources, Writing – review & editing. JD: Conceptualization, Data curation, Investigation, Methodology, Project administration, Resources, Visualization, Writing – review & editing. JY: Conceptualization, Formal analysis, Methodology, Supervision, Visualization, Writing – original draft, Writing – review & editing.
